# Entropy Analysis of High-Definition Transcranial Electric Stimulation Effects on EEG Dynamics

**DOI:** 10.3390/brainsci9080208

**Published:** 2019-08-20

**Authors:** Diego C. Nascimento, Gabriela Depetri, Luiz H. Stefano, Osvaldo Anacleto, Joao P. Leite, Dylan J. Edwards, Taiza E. G. Santos, Francisco Louzada Neto

**Affiliations:** 1 Institute of Mathematical Science and Computing, University of Sao Paulo, Sao Carlos 13566-590, Brazil; 2 Ribeirao Preto Medical School, University of Sao Paulo, Ribeirao Preto 14049-900, Brazil; 3 Moss Rehabilitation Research Institute, Elkins Park, PA 19027, USA; 4 School of Medical and Health Sciences, Edith Cowan University, Joondalup, WA 6027, Australia

**Keywords:** complexity measure, approximated entropy, time series, biosignal

## Abstract

A foundation of medical research is time series analysis—the behavior of variables of interest with respect to time. Time series data are often analyzed using the mean, with statistical tests applied to mean differences, and has the assumption that data are stationary. Although widely practiced, this method has limitations. Here we present an alternative statistical approach with sample analysis that provides a summary statistic accounting for the non-stationary nature of time series data. This work discusses the use of entropy as a measurement of the complexity of time series, in the context of Neuroscience, due to the non-stationary characteristic of the data. To elucidate our argument, we conducted entropy analysis on a sample of electroencephalographic (EEG) data from an interventional study using non-invasive electrical brain stimulation. We demonstrated that entropy analysis could identify intervention-related change in EEG data, supporting that entropy can be a useful “summary” statistic in non-linear dynamical systems.

## 1. Introduction

Following Prigogine [1], entropy is a measurement of complexity, among time series (TS) or signal data, which associates the amount of information to a probability distribution. Before the 1990s, given computational technological constraints, early entropy measure calculations were neglected because they required great amount of data [2,3].

Time series data could be defined as observed values in time order. An illustrative example of entropy motivated by Pincus’ work [4] can be expressed in two simple time series. The first is perfectly regular, alternating between 0 and 1, such as 0,1,0,1,0,1,0,1,0,1,…, whereas the second is constructed by randomly drawing 0 and 1 with probability 1/2 each, for example 0,1,0,0,1,1,0,1,1,1,0,1,0,1,1,0,…. Moments of this example, such as mean and standard deviation, will not distinguish them because both series have mean and deviation equals to 1/2, respectively. However, the first is a periodic time series, while the second is not.

In the medical field, most of the data that is acquired as time series is modeled through its mean (may also be complemented with its variance) that enables the application of the usual statistical methods. According to the definition, a stationary process relies on the unconditional joint probability distribution time-invariant. Therefore, stationarity is required to get a proper summarization of a process via its mean, and this often is not the case in real data with temporal dependence.

Statistical analyses of time series require methods to incorporate the description of all moment-generating function associated with the stochastic process, with the purpose of differing regularity from chaos in data. Entropy has been used to describe the changes in gene expression [5], cardiac signals [4], postural control sway [6], blood oximetry [7], and to characterize epileptic seizure using electroencephalographic (EEG) data [8]; however, much more could be explored using complex outcome measures.

Nonlinear dynamical analysis is commonly used to interpret biological systems, including the application of entropy for EEG analysis. Richman and Moorman [3] discuss the physiological time-series analysis towards similar epochs using entropy. Stam [9] presents a novel review about nonlinear dynamical features of information processing, such as local brain dynamics or the nonlinear synchronization between recordings from different brain regions. Moreover, Acharya et al. [10] decomposed EEG signals and extracted entropy features to identify areas affected by Epilepsy seizures.

The objective of the present study was to apply a robust methodology into a biological data set, considering its complex structure in the statistical estimation processes. The nature of the EEG data where brain electrical connections are present indicates that entropy form is a suitable method of analysis. In this context, the current study is innovative since it combines the entropy method with the appropriate statistical analysis, and mixed effects models due to the presence of unobserved variables. This work is divided into two parts, the statistical robustness discussion, and the clinical gain by its adoption.

The present data set used to describe the statistical approach was originated from a randomized double-blinded sham-controlled clinical trial that aimed to investigate a polarity and intensity-dependent shift in high-density EEG signal following an intervention using high-definition transcranial direct current stimulation applied over the temporoparietal junction in healthy participants (for protocol details read [11]).

We adopted two different statistical methods related to entropy for summarizing time series as a single number; the Kullback-Leibler (KL) divergence and the Approximate Entropy (ApEn). Both incorporated all the generated moments of the process, and enabled the use of traditional models such as regression, while the independence assumption on the observations was not violated. Consequently, it was possible to compare regularity contained in data, taking into account computational feasibility, without losing valuable information. The Approximate Entropy was introduced in the medical field by Pincus [4] as a method to discriminate biosignals events. Its first application aimed to analyze the difference between healthy and altered patterns of heartbeat in infants, through its electrocardiograms (ECGs), since it was known that patients with heart disease have more regularity in the heartbeats. We now investigate its applicability in the context of Neuroscience.

The Kullback-Leibler (KL) divergence is the most commonly presented in statistics, and the Approximate Entropy (ApEn) is described as useful in the case of non-stationary time series. This application in experimental neurology relies on an established technique of transcranial stimulation, indicated as a powerful experimental tool over the last two decades; despite the few reproduced studies reported in the literature [12]. Our research findings integrate the knowledge for the transient induction of vertical misperception in healthy subjects using electrical stimulation, and consolidates the results found by Santos et al. [11] using usual statistical methods.

## 2. Methods

### 2.1. Overview

The paper is organized as follows. In the Methods Section 2, we included the background theory (Section 2.2), where we present an overview of the effects and a brief description of the experimental protocol, and the concepts behind the definition of entropy are adequately described. In Section 2.3 (“Simulation”), we use numerical simulations to establish a comparison between representing a TS process using its mean versus its entropy, in a controlled scheme. As the results (Section 3), we apply the use of entropy to our data analysis. We aim at an efficient summarization of our TS, using a hierarchical model to distinguish between stimulation types versus intensity, as well as quantifying the differences in regularity among them. Finally, some concluding remarks are presented in Section 4.

### 2.2. Background Theory

#### 2.2.1. Transcranial Direct Current Electrical Stimulation

Transcranial direct current stimulation (tDCS) is a non-invasive neuromodulation technique that delivers a weak electrical current to the brain using electrodes attached to the scalp. While the conventional protocols of tDCS use two sponge electrodes and stimulate a large area of the brain, the high-definition protocols use multiple (>=4) and smaller gel-based electrodes that reach a more focused brain area. Depending on the electrical current polarity (among other factors), the direct effects of tDCS include the change in neuronal excitability [13]. Generally, cathodal stimulation induces a decrease in neuronal excitability, whereas anodal stimulation induces an increase in cortical excitability [14,15].

In addition to the direct tDCS effects, there are “indirect” effects driven by the connections of the targeted area with distant cortical and sub-cortical areas [16]. Using functional magnetic resonance imaging, Lang et al. [17] showed that the tDCS application over the right primary motor cortex (M1) activates not only the areas under the electrodes but also several connected brain areas. These results were interpreted based on the physiological concept of the functional interaction between M1 and the diverse activated areas via cortico-cortical and cortico-subcortical connections. Other studies using transcranial magnetic stimulation (TMS) described the increased activity of the homologous area, contralateral to the stimuli [18,19,20,21]. Therefore, to assess the physiological effects of tDCS, it is recommended to investigate not only under the targeted brain area but also in distant areas of the related neural network.

Noninvasive techniques of brain stimulation are current therapeutic resources related to the pathophysiology and behavior of the mechanisms that guide the human mind. In the field of neuroscience, the clinical application of these tools has gained greater repercussion in the last two decades, resulting in an eventual increase in the number of studies and clinical trials in this area [22,23]. Several studies have indicated the therapeutic efficacy of the use of non-invasive stimulus in psychiatric, neurological, and motor disorders [16,24,25,26]

The influence of transcranial direct current electrical stimulation protocols in human postural control has been described previously [27,28]. However, even with current evidence and decades of experimentation on transcranial direct current stimulation, few protocols have achieved robust scientific acceptance. Among the open questions about tDCS in humans, the dose-response effects can be highlighted to develop more effective protocols for rehabilitation.

#### 2.2.2. Stimulation Protocol

**Figure 1:**
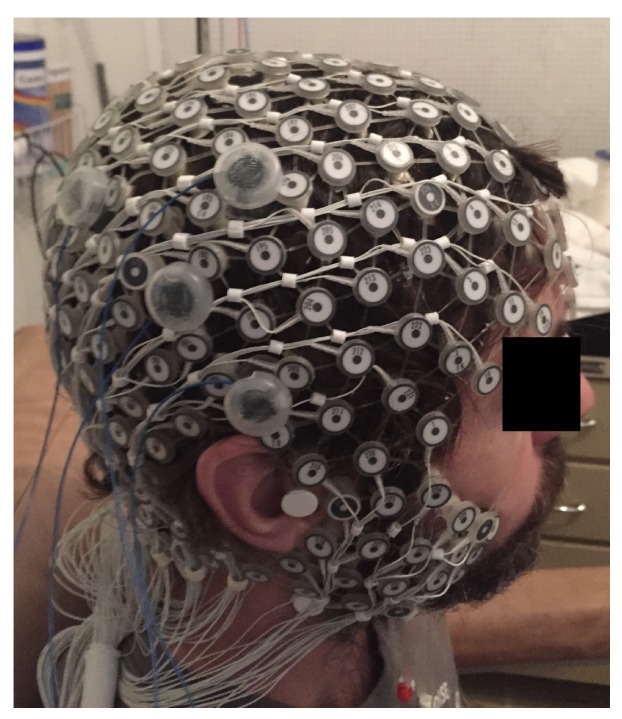
Position of the HD-tDCS electrodes and EEG 256-channel sensor net in a male participant. Written informed consent was obtained from the participants for the publication of this image.

The study protocol, published in detail elsewhere [11], aimed to systematically analyze the dose-response effects of a focal electrical stimulation. The HD-tDCS was placed over the right temporo-parietal junction (TPJ) region (Figure 1). Seven healthy right-handed participants, mean age 34.7±7.6 years, four men were assessed. A Soterix^®^ NY-USA equipment of HD-tDCS was used, with a constant current from the anode to cathode. Participants received three electrical stimulation conditions (anode center, cathode center, and sham) on three different days, with an interval of at least 24 h.

The $HD-tDCS_{3\times 1}$ montage included four electrical stimulation electrodes; the single center electrode was placed on the right hemisphere in the circumcenter of a triangle with vertices on the EEG coordinates C4, T8, P4. The three surround electrodes were placed at a distance of 3 cm from the central electrode, over P4, C4, and T8. The same position of the electrodes was used during the sham condition. In the beginning of every session, an accommodation protocol was applied to increase tolerability for participants, with the intensity of stimulation varying from 1 to 3 mA. The stimulation protocol comprised 3 blocks of 2 minutes at each current intensity (1, 2, and 3 mA), and an inter-stimulus interval of 5 minutes between blocks. The approximate total duration of each session was 120 min.

A dense array EEG with 256-channel sensor net (Electrical Geodesics Inc., Eugene, OR, USA) with a sampling frequency of 500 Hz (observation points per second) was used to detect ongoing changes on the cortical neuronal activity in response to HD-tDCS. All channels were referenced to the vertex (electrode CZ) with electrical impedance reduced. The EEG was recorded continuously before and after the stimulation, including ramp-up and ramp-down periods, lasting in total between 120 min. The data analyzed in the present study corresponds to the interval between the stimulation periods.

#### 2.2.3. Entropy Background

As an alternative to analyzing and modeling the entire TS, one can use summary statistics, which could be the average of the processor, for example, some measure of its regularity. The last one is commonly used as an index to quantify the complexity of the TS, considering all of its generated moments. It is relevant to discuss the relation between maximum likelihood estimation (MLE) and information entropy.

Consider a random sample $X=\{X_{1},\cdots ,X_{n}\}$ of random variables, all with a common (but unknown) density $f(x|\theta_{0})$. From the data, it is possible to estimate its associated unknown parameter θ, and then associate to it a density function (pdf) family (e.g., normal, gamma, beta etc.). Our goal is to estimate θ through a robust statistic *T*, using the data $X_{1},\cdots ,X_{n}$, that is, $\hat{\theta }=T\left(X_{1},\cdots ,X_{n}\right)$. As an illustration of a statistic we could have, for example, the sample mean, in the case the times series under analysis are stationary,

$$T\left(X\right)=\bar{X}=\frac{1}{n}\sum_{i=1}^{n}X_{i}.$$

Then, using the MLE, one could obtain $\hat{\theta }$ as a joint density of the random sample, and using a traditional result known by the (Strong or Weak) Law of Large Numbers, the sample negative log-likelihood converges to its expected value (almost surely or in quadratic mean). Moreover, by the asymptotic equipartition theorem (further details in McMillan [29]), this will converge to the differential entropy of *X*. If *x* denotes the sample observations, then

$$h\left[f\left(x|\theta_{0}\right)\right]=-E\left[\log f\left(X|\theta_{0}\right)\right]=-\int_{R}f_{X}\left(x|\theta_{0}\right)\log f_{X}\left(x|\theta_{0}\right)dx$$

Notice that using the expectation of the parameter, does not fix θ at $\theta_{0}$, and does not give us any information about the conversion of the sample negative log-likelihood, and how it should behave (the only assumption of the asymptotic equipartition theorem is related to $\theta_{0}$). Therefore, adding and subtracting the log-likelihood under the true model,

$$\begin{array}{c} -\frac{1}{n}\sum_{i=1}^{n}\left[\log f\left(X_{i}|\theta \right)+\log f\left(X_{i}|\theta_{0}\right)-\log f\left(X_{i}|\theta_{0}\right)\right]=\\ =\frac{1}{n}\sum_{i=1}^{n}\log\left[\frac{f(X_{i}|\theta_{0})}{f(X_{i}|\theta )}\right]-\frac{1}{n}\sum_{i=1}^{n}\log f\left(X_{i}|\theta_{0}\right)=\\ =f\left(x|\theta_{0},\theta \right)-f\left(x|\theta_{0}\right) \end{array}$$

Now the divergence of this estimator will be denoted as

$$D_{KL}(f\left(x|\theta_{0}\right)||f\left(x|\theta \right))=h\left[f\left(x|\theta_{0},\theta \right)\right]-h\left[f\left(x|\theta_{0}\right)\right]$$

where

$$D_{KL}(f\left(x|\theta_{0}\right)||f\left(x|\theta \right))=\int_{R}f\left(x|\theta_{0}\right)\log\frac{f(x|\theta_{0})}{f(x|\theta )}dx$$

known as the Kullback-Leibler divergence or relative entropy between $f(x|\theta_{0})$ and f(x|θ). Notice that the mean negative log-likelihood converges to the differential entropy under the true distribution plus the Kullback-Leibler divergence between the true distribution and the assumed distribution.

It is also possible to show that the Kullback-Leibler divergence is non-negative and is zero only when $f(x|\theta_{0})$ = f(x|θ) almost surely. Consider that to minimize the mean negative log-likelihood, implies that it is needed to choose means $\theta =\theta_{0}$, which minimizes this limiting function.

Moreover, after a bit of a detour through information theory, we have seen a sketch as to why MLE makes sense as a procedure for estimating a parameter θ. The mean negative log-likelihood converges to a non-random function, as so takes its minimum at the correct answer to our question. It is fully proving the consistency of the Maximum Likelihood Estimator it is out of this work scope.

Limitation among regularity in TS data is presented among the usage on entropy, where stationarity is also recurrently needed. Despite this fact, the regularity statistic suggested by Pincus [2] uses the capability to discern the changing of the complexity from such a relatively small amount of data.

Simplistically, the proposed solution to summarize the TS, in one representative statistic, is based on a recurrent calculation of conditional probabilities at the i-th time-window. The result is an average obtained from the numbers of distance superior to the filter (r), $d\left[x,x^{*}\right]=\max\left|u\left(a\right)-u^{*}\left(a\right)\right|$, therefore calculating through $C_{i}^{m}\left(r\right)$ as a relative frequency of generated vector.

Let us consider the vector *u* a vector of collected data, u(i) is the *i*-th observation of *u*, and *x* is an element of a partition as following. Considering x(1)={u(1),…,u(m)},x(2)={u(m+1),…,u(2m)}, …,x(N−m+1)={u((N−m)m+1),…,u((N−m+1)m)}.

STEP 1. Consider a TS, equally spaced in time, contained *N* raw data values (*u*), from each series. Then *u(1), u(2), …, u(N)*.

STEP 2. Adopt a length of compared runs of data (*m*), and a filtering level (*r*). Implying in a new vector of data *x(1), x(2), …, x*(N−m+1).

STEP 3. For each *i*, 1<i<N−m+1, to construct $C_{i}^{m}\left(r\right)=\left(cardinal\;d\left[x\left(i\right),x\left(j\right)\right]\leq r\right)/\left(N-m+1\right)$ where i≠j

STEP 4. Then, $\Phi^{m}\left(r\right)=\sum_{i=1}^{N-m+1}\ln C_{i}^{m}\left(r\right)/\left(N-m+1\right)$

STEP 5. Calculate the average over i of ln towards conditional probability as $ApEn=\Phi^{m}\left(r\right)-\Phi^{m+1}\left(r\right)$

The choice towards *m* affects the conditional probabilities directly well achieved with between $10^{m}$ and $30^{m}$ observations (as defined by Pincus [2]). The filter level (r) suggested is at least three times the estimated mean noise amplitude. Important to point out about the consistency, even ApEn not been an absolute measure, theoretic analyses, whenever entropy(A)≤entropy(B) for noiseless systems, then ApEn(A)≤ApEn(B).

### 2.3. Simulation

The mean estimator could be a good statistic to summarize a collection of the observation given some mild behavior, e.g., in TS under the presence of stationarity. However, this is often not the case in medical data. In order to illustrate a simulated case, four models were considered as follows, which 100 simulated series of each was calculated then it is mean and Approximated Entropy were estimated, considering different sample size (n) as n={70,100,250,500,1000}. Whereas Autoregressive (AR) model and Generalized AutoRegressive Conditional Heteroskedasticity (GARCH) model.

$$\begin{array}{cc} Y_{t}^{\left(1\right)}= & \;\phi_{1}Y_{t-1}+\epsilon ,\;\;\;\;\left[AR\left(1\right)\right]\\ Y_{t}^{\left(2\right)}= & \;\gamma_{0}+Y_{t}^{\left(1\right)}+\alpha_{0}+\alpha_{1}\epsilon_{t-1}^{2}+\beta_{1}h_{t-1}^{2},\;\;\;\;\left[Inter.+AR\left(1\right)+GARCH\left(1,1\right)\right]\\ Y_{t}^{\left(3\right)}= & \;\gamma *t+Y_{t}^{\left(1\right)},\;\;\;\;\left[Linear\;Trend+AR\left(1\right)\right]\\ Y_{t}^{\left(4\right)}= & \;\frac{\gamma *t^{2}}{1000}+Y_{t}^{\left(1\right)}+\alpha_{0}+\alpha_{1}\epsilon_{t-1}^{2}+\beta_{1}h_{t-1}^{2},\;\;\;\;\left[Quadratic\;Trend+AR\left(1\right)+GARCH\left(1,1\right)\right] \end{array}$$

where $\phi_{1}=0.7,0.9$ as the autoregressive with different parameters, $\gamma_{0}=2$ as an intercept, γ=0.02,0.2 as parameters for the deterministic trends (linear and quadratic). The γ parameter gives the series the strength (influence) of its deterministic part.

**Figure 2:**
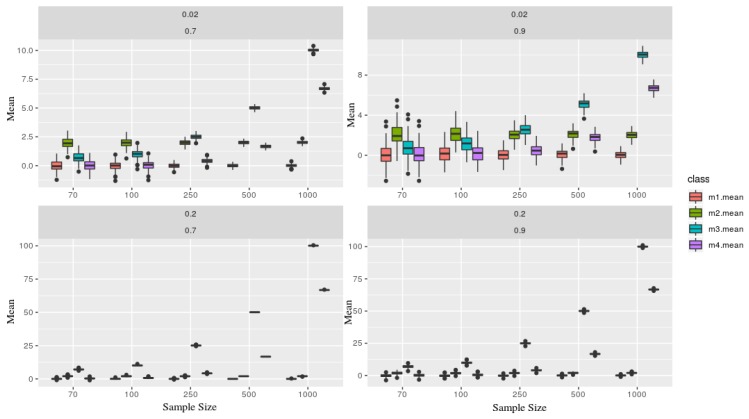
Each figure represents a simulated series, which contains two parameters γ=0.02,0.2 and ϕ=0.7,0.9 (in the top of each board). Each class (color) is associated with a given model, where as the sample size (x-axis), and y-axis the **mean** statistic. Models x1 and x2 are presents more stochastic components than x3 and x4, therefore one can not recognize through its means.

As expected, Figure 2 shows that the mean estimator is very sensible, given where the series sample was taken. Although, primarily, they are all generated from the same AR(1). As the sample size grows, both trends influence more on its estimation.

Considering the EEG montages (set), a standard methodology is to use a *common reference montage* which compare every electrode in the head against a referenced one (usually the central, Cz). Therefore, each participant will present a different unit amplitude wave, regardless of the channel, given this recording methodology uses a central channel to take an amplitude difference as a reference. Then becoming extremely sensitive to each participants’ characteristic, so it can be bypassed using, for example, an Approximate Entropy.

**Figure 3:**
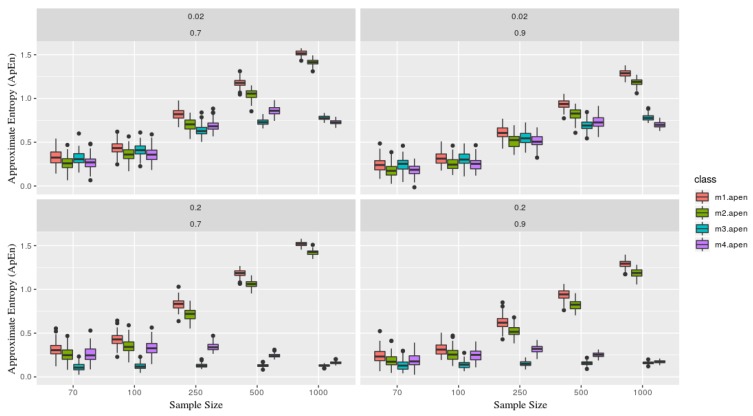
Each figure represents a simulated series, which contains two parameters γ=0.02,0.2 and ϕ=0.7,0.9 (in the top of each board). Each class (color) is associated with a given model, where as the sample size (x-axis), and y-axis the **Approximate Entropy** statistic. Models x1 and x2 are presents more stochastic components than x3 and x4, now recognize based on its entropy, especially as the sample size grows (showing stability). That is, x1 and x2 gets close to 1, meaning the is predominately originated from a stochastic process.

Fixing the parameters, for the entropy calculation, considering m=2 and r=0.2. Figure 3 explicit the simulated scenarios. Upper panels consider γ=0.02 and $\phi_{1}=0.7,0.9$ showing stability for models m3 and m4 (the ones containing deterministic part) versus m1 and m2 as the sample size grows. Lower panels consider γ=0.2 which describes the increase of the deterministic part in models m3 and m4, and this implies on keeping ApEn close and a low range regardless of its sample size process (that is, closer to zero, which presents a great deterministic component in the series).

It is essential to mention, as Pincus [2] elucidates, that ApEn stability it is conditioned to the fixed parameter *m* where the number of observations (n) needs to be between $10^{m}<n<30^{m}$. Thereby, samples below this will not guarantee its efficiency.

The influences observed in the estimations of the mean exemplifies this statistic is non-robust to under non-stationarity TS. Dealing with EEG signal is challenging, given its scale conditional to its participant and brain region. The record is made using the technology of a differential amplifier, which takes the difference of two inputs and displays only one output, as their difference, useful to small electrical impulse systems. EEG signals are calculated based on personal reference, therefore, they are not absolute across subjects. Additionally, this data may become too noisy.

## 3. Results

In order to test the intensity and polarity-dependant effect of HD-tDCS, we designed [11] an experiment in which we compared the effect Anodal and Cathodal stimulation protocols in different phases (baseline, 1 mA, 2 mA, and 3 mA). Data was acquired via Electroencephalogram technique, and then its time series process was summarized using entropy indices.

The dynamic across the brain network connectivity is a complex phenomenon of substantial relevance which could help neurologists to understand better some diseases and help on the development of new treatments. Different brain areas could be compared using complexity as a measurement among neural network integration.

Our results are divided into two parts. First, we illustrate one single case, to compare brain regions activation (as channels) using relative entropy, and then we consider the entire dynamic of the experiment (dose-response versus conditions). Both parts used responses from 51 EEG channels located over the TPJ cortical region.

### 3.1. Analyzing Complexity within Channels

We propose an information theoretic approach to neuroscience, with the application of the Kullback-Leibler divergence and the Approximate Entropy to the analysis of time series. Our goal is to compare different processes taking into account all their distribution moments (considering much more than only their average), thus enabling further analysis between neural events [30,31,32,33].

To illustrate the previous discussion, let us consider the *Kullback-Leibler* divergence (KL) between channels. For simplicity, we examined just a single trial of one participant, giving the possibility to analyze the process’ synergy difference between the 51 channels located in the central region (left and right motor brain area). All the analyzes consider the smoothed TS, that is, with one observation point per the second resolution, with only 300 observations per phase (dose-response).

Let us bear in mind that the KL divergence is not symmetric. To make it asymmetric distance measure, we use the average shown below,

(1) $$KL_{DIST}\left(p,q\right)=\frac{KL(p,q)+KL(q,p)}{2}.$$

In our case, the probability distributions represent electrical activity from each EEG channel.

**Figure 4:**
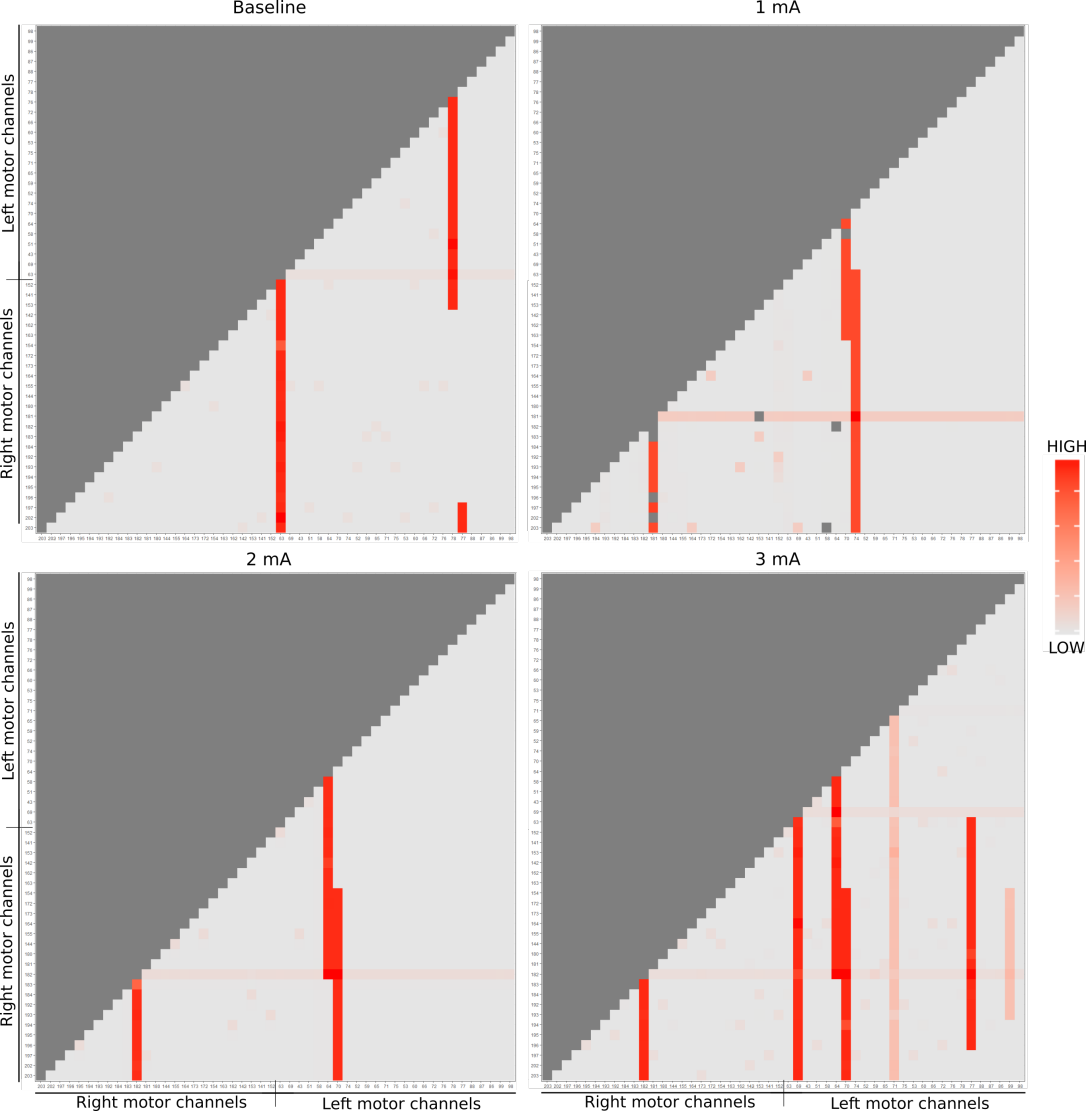
EEG entropy analysis of dose-response are represented by each matrix. Due to the matrix symmetry, it was considered only the lower triangular part (light gray). The red color scale correlates the energy dynamics synergy of each EEG channel, being the dark red of greater intensity and the light of lesser.

In Figure 4, we use the pairwise complexity measurement (1). Due to the imposed symmetry, it suffices to visualize only the bottom right to perform a comparison among the channels through the brain hemisphere (25 first channels refer to the left brain side [left motor + left temporal] and right respectively). The imposed symmetry is particularly crucial because we needed to know if, across each intervention, there exists a change in the dynamics of system complexity (if one stimulation region is impacting/connected to another). As well, this helps in describing the activation areas and its structural relations.

Notice that during baseline state (top left figure), channel 63, placed in the right side motor, reacted remarkably, as well as channels 77 and 78 with some other channels. Due to the electrical stimulus at 1mA (top right figure), it is possible to notice some change in the dynamic according to the stimulation regions: especially channels 181 (stimulation tDCS point), 70 and 74 (the equivalent points in the other hemisphere) present a larger entropy. In the 2 mA stimulation (bottom left figure), the activity in channels 182, 70, and 64 (the neighbor of the stimulation tDCS point and the equivalent points in the other hemisphere) is highlighted. Finally, in the 3 mA (bottom right figure), the channels 182, 69, 64, 70, and 77 are emphasized concerning the others, while channels 71 and 99 also point out, but present smaller statistics.

Other space reduction approaches are presented in the literature. For instance, space reduction and concurrent were used to describe the interference effects of transcranial magnetic stimulation (TMS) on several electroencephalographic (EEG) measures providing causal evidence towards a microstates modification [34,35]. Moreover, estimated subspaces are created to represent different brain activity patterns belonging to the dataset. Therefore, this approach enables the comparison between brain regions in each dose-response, by summarizing its complexity. In the case of a non-stationary process, the class of entropy must take this fact into account.

### 3.2. Analyzing Complexity across Dose-Response Effect

Pincus [4] presented the so-called *Approximate Entropy* (ApEn) as a technique to quantify the amount of regularity, and the unpredictability of fluctuations, over time-series data not conditional to its stationarity. This entropy is particularly interesting for this application, given the main questions are settled discerning about the regularity of the dose-response across the montages. Therefore, aiming to test the patient response, the entropy approach target is to summarize the comparison towards the induced neuromodulation, that is, the electrical stimulation.

Given the presence of repetitive patterns, in a time series, its predictability renders conditionally to its fluctuation. ApEn can be interpreted as the likelihood that similar patterns of observations will not be followed by additional similar observations, calculated by

$$ApEn=\Phi^{m}\left(r\right)-\Phi^{m+1}\left(r\right),\;where\;\Phi^{m}\left(r\right)={\left(N-m+1\right)}^{-1}\sum_{i=1}^{N-m+1}ln\left(C_{i}^{m}\left(r\right)\right)$$

where *N* is raw data values from a equally spaced in time, *m* is the length of compared time-window data, *r* is filtering level and $C_{i}^{m}\left(r\right)$ measures within a tolerance *r* the regularity of patterns similar to a given pattern.

**Figure 5:**
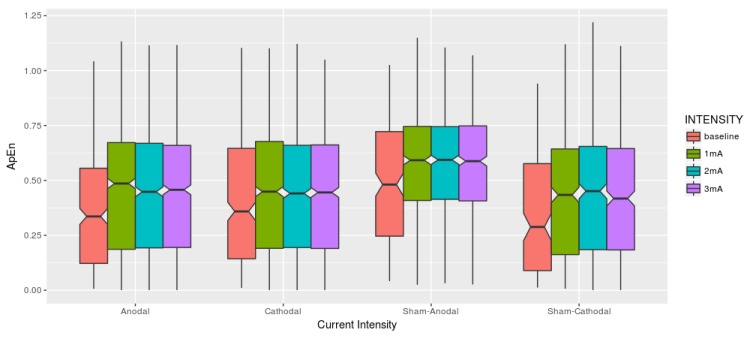
Each current intensity is related to one condition (Anodal, Cathodal, Sham-Anodal, Sham-Cathodal). Visually the boxplots seem to present equivalent results among the brain stimulation conditions also considering the different intensities.

In this manner, m=2 and r=0.2×sd(TS) were adopted as parameters, and the ApEn was used to summarize the time series block experimentation and then adjusted a mixed-effect model in other to verify its similarity. A Time Series which contains many repetitive patterns has a relatively small ApEn; a less predictable process has a higher ApEn. Figure 5 describes the fluctuations over time-series data in each stimulation condition.

Complementing the descriptive analysis, Table 1 brings the basics statistical descriptive under the optic of the Approximate Entropy among dose-response within Conditions. It is to be noticed that both mean and median increased once the stimulus is applied although remaining quite similar among them, sharing a similar range of standard deviation. One thing we would like to highlight is that the 3 mA intensity in the Cathodal condition has the smallest maximum ApEn.

The theoretical adopted mixed model is explicit by Equation (2), represented as

(2) $$\begin{array}{c} Y=X\beta +Z\gamma +\epsilon \end{array}$$

where the Y is a vector of the ApEn containing 51 channels, per participant, X and Z are design matrices, β refers to the fixed-effects related to the intercept, montage (stimulation), intensity and their interaction, and γ is a matrix including the random-effects been channel and condition nested participant.

The number associated with the intention stage (configured by each dose-response—1, 2 and 3 mA) is equivalent to ten periods in total (baseline + 3 replicas of each dose-response). Due to the number of the condition is three (Anodal, Cathodal, and Sham), per participant which are seven, then twenty-one obtained trials. All this combination summarizes in a total of 10,710 observations (given that a scalar number—ApEn represents each TS). Therefore, the model’s fixed effects were converted into dummies variables (Stimulations versus Intensity).

According to Table 2, which presents the estimations relating the variances of each random component, a similar variance among the different stimulation condition is observed conditioned to the participants (involving personal characteristic).

Analyzing the estimates associated with the fixed effects (Table 3) and considering a significance level of 5%, there were differences between the complexity across the current intensity effects (without discriminating the stimulation condition). No difference was shown across stimulus (regardless of electrical stimulation) using the Anodal stimulus as a reference to compare the conditions. Then, observing the iteration among stimulus versus intensity, only Cathodal with 2 mA presented to be statistically different between condition (Anodal vs. Cathodal).

The method presented here contributes to the findings described by Santos et al. [11] that described results bypassed through non-parametric techniques considering only channels 164 and 66, now extended to a larger brain region composed mainly by 51 channels. In the previous work [11], the Kruskal-Wallis test also revealed intensity-dependent effects on the cathode center condition in the gamma frequency band, and the Tukey post-hoc test indicated a significant difference between 2 and 3 mA (*p* = 0.044).

Furthermore, Table 4 presents the correlation of fixed effects matrix, consider Sham (S), Anodal (A) and Cathodal (C).

The present results support the observations of Santos et al. [11], despite the differences between the analyses, where two (among our 51) channels were considered, and 5 s (against our 5 min) of post stimulation EEG recording were investigated. The results presented in this subsection agree with Bikson et al. [36] and Perennou et al. [37], where the importance of a safe protocol is highlighted.

## 4. Conclusions

This work aimed to present and discuss the use of an appropriate statistic to summarize time series processes, preserving the information contained therein. Thus, entropy was suggested as a robust alternative replacing the average since the processes observed in real life do not show stationarity. In the medical field, traditional statistical models are commonly adopted, which generally depart from the principle of independence between data, then time series are pre-processed (summarized) in an attempt to fit data into these types of models.

Entropy is a critical “summary” statistic in nonlinear dynamical systems analysis and chaos. By using entropy, in the area of neuroscience, it has a straightforward interpretation which is associated with the energetic dynamics of the process then statistical hypothesis tests comparing their equivalences. Moreover, in this study, under this approach, we could discuss the feasibility of the protocol [11], and its safety, towards treatment as a vertical human manipulation task presented in, e.g., post-stroke patients.

This work is shown the Kullback-Leibler divergence as a space reduction approach, representing the variation across EEG channels (located in different brain areas), and targeting to explicit the electrical dynamic from a single participant (in Figure 4). Then, analyzing the entire clinical experiment, elucidated by protocol [11], using a nonlinear dynamic analysis through Approximate Entropy and considering not only two channels (164 and 66), but extending the analysis using all the 51 channels from a larger cortical region. Moreover, relative direction and structural change with stimulation intensity towards cathode center condition among the dynamic between baseline and its current intensities were suggested.

Future studies should explore the time-varying parameters dynamic across stimulus (intensity) versus conditions (polarity/sham). Other approaches could be further explored adapting different metrics, such as Mahalanobis or Geodesic distance, in the complexity calculation used in the approximate entropy measure, as well as incorporate a time-varying filter level parameter.

## Figures and Tables

**Table 1:** ApEn summary among Stimulus versus Intensity.

Condition	Intensity	MEAN	SD	MIN	MEDIAN	MAX
Anodal	base	0.365	0.260	0.0064	0.336	1.043
	1 mA	0.452	0.280	0.0004	0.486	1.133
	2 mA	0.445	0.278	0.0006	0.448	1.115
	3 mA	0.444	0.279	0.0006	0.457	1.116
Cathodal	base	0.397	0.284	0.0108	0.359	1.104
	1 mA	0.447	0.281	0.0006	0.449	1.101
	2 mA	0.433	0.276	0.0006	0.441	1.121
	3 mA	0.440	0.273	0.0006	0.445	1.050
Sham	base	0.430	0.283	0.0121	0.418	1.026
	1 mA	0.512	0.264	0.0068	0.536	1.150
	2 mA	0.514	0.260	0.0005	0.534	1.220
	3 mA	0.510	0.259	0.0008	0.532	1.112

**Table 2:** Mixed model—Random effects estimations.

Groups		Variance	Std.Dev.
Channel	(Intercept)	0.0012	0.0348
Key	Anodal	0.0119	0.1089
	Cathodal	0.0113	0.1063
	Sham	0.0165	0.1283
Residual		0.0634	0.2519

**Table 3:** Mixed model—Fixed effects estimations.

	Estimate	Std. Error	t Value	*p*-Value	
(Intercept)	0.365	0.044	8.373	5.13e-05	***
INTENSITY 1 mA -Baseline	0.088	0.015	5.706	1.19e-08	***
INTENSITY 2 mA -Baseline	0.080	0.015	5.221	1.82e-07	***
INTENSITY 3 mA -Baseline	0.079	0.015	5.153	2.61e-07	***
CONDITION Sham-Anodal	0.066	0.052	1.266	0.2425	
CONDITION Cathodal-Anodal	0.033	0.044	0.735	0.482	
INTENSITY 1 mA:Sham-Anodal	−0.006	0.022	−0.298	0.7656	
INTENSITY 2 mA:Sham-Anodal	0.003	0.022	0.137	0.8913	
INTENSITY 3 mA:Sham-Anodal	0.000	0.022	−0.006	0.9951	
INTENSITY 1 mA:Cathodal-Anodal	−0.038	0.022	−1.730	0.0837	.
INTENSITY 2 mA:Cathodal-Anodal	−0.044	0.022	−2.029	0.0425	*
INTENSITY 3 mA:Cathodal-Anodal	−0.037	0.022	−1.693	0.0905	.

—Signif. codes: 0 ‘***’ 0.001 ‘**’ 0.01 ‘*’ 0.05 ‘.’ 0.1 ‘ ’ 1.

**Table 4:** Mixed model—Correlation of fixed effects estimations.

	(Intr)	1mA	2mA	3mA	S:A	C:A	S:A1mA	S:A2mA	S:A3mA	C:A1mA	C:A2mA
1mA	−0.265										
2mA	−0.265	0.750									
3mA	−0.265	0.750	0.750								
S:A	−0.461	0.222	0.222	0.22							
C:A	−0.721	0.260	0.260	0.26	0.535						
S:A1mA	0.187	−0.707	−0.530	−0.53	−0.314	−0.184					
S:A2mA	0.187	−0.530	−0.707	−0.53	−0.314	−0.184	0.75				
S:A3mA	0.187	−0.530	−0.530	−0.707	−0.314	−0.184	0.75	0.75			
C:A1mA	0.187	−0.707	−0.530	−0.53	−0.157	−0.368	0.5	0.375	0.375		
C:A2mA	0.187	−0.530	−0.707	−0.53	−0.157	−0.368	0.375	0.5	0.375	0.75	
C:A3mA	0.187	−0.530	−0.530	−0.707	−0.157	−0.368	0.375	0.375	0.5	0.75	0.75
